# Comparison of ultrasound- and computed tomography-guided pulsed radiofrequency in treating ophthalmic branch postherpetic neuralgia: a retrospective study

**DOI:** 10.1590/1806-9282.20241004

**Published:** 2024-11-11

**Authors:** Qianqian Shen, Bo Wang, Jianmin Yu, Jurong Xia

**Affiliations:** 1 Hangzhou Third People's Hospital, Department of Nursing and Dermatology – Hangzhou, China.; 2 Hangzhou Third People's Hospital, Department of Anesthesiology and Pain Medicine – Hangzhou, China.

**Keywords:** Ophthalmic nerve, Postherpetic neuralgia, Pulsed radiofrequency, Ultrasound, CT

## Abstract

**OBJECTIVE:**

The objective of this study was to compare the efficacy and safety between ultrasound- and computed tomography-guided pulsed radiofrequency in treating ophthalmic branch postherpetic neuralgia.

**METHODS:**

A retrospective study was conducted on data of 84 patients with ophthalmic branch postherpetic neuralgia. According to the puncture guiding method, the patients were divided into the ultrasound- and computed tomography-guided groups, which received the ultrasound- and computed tomography-guided supraorbital nerve pulsed radiofrequency treatment, respectively. The puncture time, numeric rating scale pain score before pulsed radiofrequency and after pulsed radiofrequency, effective rate of treatment, and intraoperative and postoperative adverse events were observed.

**RESULTS:**

The puncture time in the ultrasound-guided group was significantly shorter than that in the computed tomography-guided group (p<0.05). At 1, 4, and 12 weeks after pulsed radiofrequency, in two groups, the numeric rating scale pain score was significantly lower than that before pulsed radiofrequency, respectively (p<0.05). At each time, the numeric rating scale pain score showed no significant difference between the two groups (p>0.05). At 12 weeks after pulsed radiofrequency, there was no significant difference in the effective rate of treatment between the two groups (p>0.05). During the intraoperative and postoperative periods, the incidences of adverse event hematoma and oculocardiac reflex in the ultrasound-guided group were significantly lower than those in the computed tomography-guided group, respectively (p<0.05).

**CONCLUSIONS:**

Both ultrasound- and computed tomography-guided supraorbital nerve pulsed radiofrequencys have good efficacy in treating the ophthalmic branch postherpetic neuralgia. Compared with the computed tomography-guided pulsed radiofrequency, the ultrasound-guided pulsed radiofrequency has faster puncture operation and is safer. It is more worthy of clinical applications.

## INTRODUCTION

Postherpetic neuralgia (PHN) may occur in the vast majority of cases of ophthalmic branch herpes zoster, which is 20 times more common than mandibular or maxillary herpes zoster^1^. The severe burning, electric shock sensation, lancinating pain, numbness, and paresthesia are the hallmarks of ophthalmic branch PHN, which have a major negative impact on the patient's quality of life^2^. For patients with PHN, relying solely on calcium channel antagonists, nerve block with steroid injection, and physical therapy with low- or high-frequency electric stimulation is insufficient^3,4^. The pulsed radiofrequency (PRF) treatment is a relatively novel pain-intervention technique. It can alleviate the pain by delivering electric impulses and heat bursts at a temperature of less than 42°C to avoid neuronal injury, contrary to the conventional radiofrequency applications that apply a constant high temperature of 60–80°C^5^. The PRF has been reported to relieve pain in certain chronic pain conditions^6^. It is found that computed tomography (CT)-guided PRF is an efficient treatment strategy for the ophthalmic branch PHN^7^. However, the CT examination is relatively expensive, and the radiation from CT scans has specific negative effects on the health of patients^8^. Compared with CT, ultrasound is less expensive and comparatively safer for human health^9^. The ultrasound has been applied to PRF for the treatment of PHN^10,11^. This retrospective study compared the efficacy and safety of ultrasound- and CT-guided PRF in treating the ophthalmic branch PHN.

## METHODS

### Patients

A total of 84 patients with ophthalmic branch PHN who received the supraorbital nerve PRF treatment in our hospital from July 2018 to November 2022 were enrolled. According to the puncture guiding method, the patients were divided into the ultrasound-guided group and the CT-guided group, with 42 patients in each group. In the ultrasound-guided group, there were 19 males and 23 females, with an average age of 68.53±8.61 years. The average disease duration was 142.41±24.20 days. There were 21 cases with the left side affected and 21 cases with the right side affected. In the CT-guided group, there were 22 males and 20 females, with an average age of 68.83±8.44 years. The average disease duration was 139.52±27.10 days. There were 19 cases with the left side affected and 23 cases with the right side affected. There was no significant difference in gender, age, disease duration, or affected side between the two groups (p>0.05) (Table 1). This study was approved by the ethics committee of Hangzhou Third People's Hospital. Written informed consent was obtained from all participants.

**Table 1:** General data of patients in two groups.

Index	Ultrasound-guided group	CT-guided group	t/χ^2^	p
N	42	42		
Gender (n)			0.429	0.513
Male	19	22		
Female	23	20		
Age (years)	68.53±8.61	68.83±8.44	0.161	0.872
Disease duration (days)	142.41±24.20	139.52±27.10	0.515	0.608
Affected side (n)			0.191	0.662
Left	21	19		
Right	21	23		

CT: computed tomography.

### Inclusion and exclusion criteria

Inclusion criteria were as follows: the age of patients was more than 18 years; the disease duration was longer than 3 months; the patients had used pregabalin for at least 2 weeks; and the numeric rating scale (NRS) pain score was more than 5 points. The exclusion criteria were as follows: The patients could not properly describe the pain to investigators; the patients had preexisting neuralgia; the patients had a history of chronic pain, systemic immune disease, or contraindications to puncture.

### Treatment procedure

**Figure 1:**
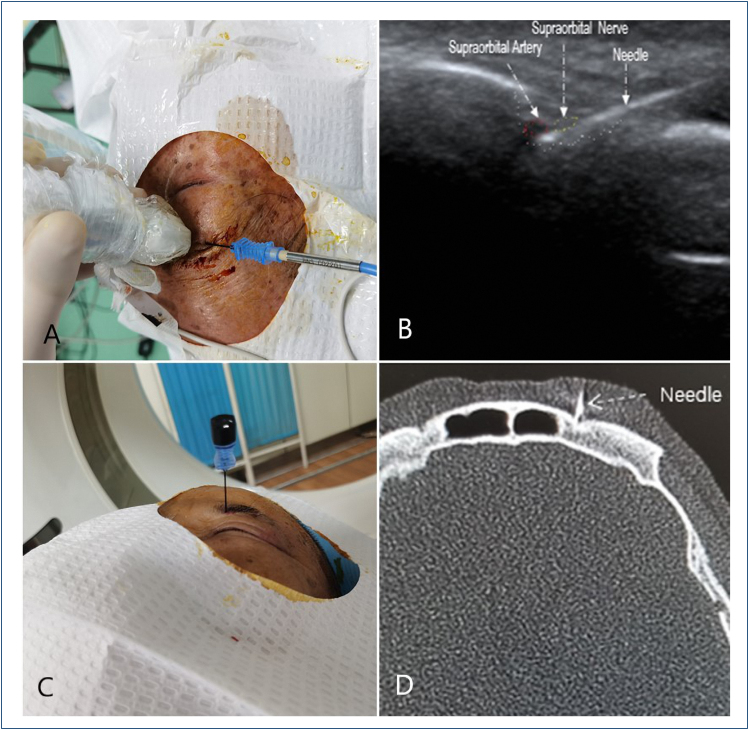
(A) Puncture operation of ultrasound-guided pulsed radiofrequency. (B) Ultrasound image of puncturing. (C) Puncture operation of computed tomography-guided pulsed radiofrequency. (D) Computed tomography image of puncturing.

Patients were positioned supine on the treatment table. The vital signs (blood pressure, respiration, pulse, body temperature, and consciousness) were closely monitored. In the ultrasound-guided group, the high-frequency ultrasonic probe (Mindray Portable Ultrasound Instrument Inc., Shenzhen, China) was placed at the eyebrow arch to show the supraorbital nerve. The PMF-22-50-4 PRF trocar (22-gauge, 5-cm electrode with a 4-mm active tip; Baylis Medical Inc., Quebec, Canada) was inserted from the outside to the inside with the in-plane method carefully until the needle tip reached below the supraorbital nerve. In the CT-guided group, the supraorbital notch or foramen on the side of the lesion was located by CT scanning. To ensure that the radiofrequency needle was positioned in the supraorbital foramen or supraorbital notch, the puncture path was designed and regularly cleaned. The radiofrequency needle was progressively inserted at a predetermined angle and depth, and the needle tip position was adjusted with the aid of CT (Figure 1). In the two groups, the RFE4 radiofrequency generator (Beijing Neo Science Co., Ltd., Beijing, China) was connected to verify the final puncture position whenever abnormal sensations (mainly soreness, numbness, thermal sensation, and an occasional twitch-like or prickly sensation) were observed over the hyperalgesia skin areas below 0.2 V. The PRF treatment was performed for a pulse time of 240 s with 3 cycles. The output voltage was set at 50 V and increased gradually to the maximum voltage (bearable without causing pain in conscious patients) with a pulse temperature of 42°C, a pulse duration of 20 ms, and a pulse rate of 2 Hz. At the end of treatment, a 1-mL mixture composed of 0.2% ropivacaine hydrochloride injection (AstraZeneca AB Inc., Stockholm, Sweden) and 1.75-mg compound betamethasone injection (Merck Sharp & Dohme Inc., NJ, USA) was used for the supraorbital nerve block.

### Observation indexes

Puncture time, NRS pain score before PRF, and at 1, 4, and 12 weeks after PRF in the two groups were recorded. At 12 weeks after PRF, the effective (the NRS score was reduced by ≥50%) cases were counted. The effective rate of treatment was calculated as follows: effective rate (%)=(number of effective cases/total case number)×100%. In addition, the intraoperative and postoperative adverse events including infection, hematoma, oculocardiac reflex, corneal injury, nerve injury, and others in two groups were recorded. The incidence of adverse events was calculated.

### Statistical analysis

The SPSS 20.0 statistical software was used to process the data. The counting data were shown as numbers or rates, and the comparison between the two groups was performed using the χ^2^ test. The measurement data were shown in mean±standard deviation. The comparison between the two groups was performed using the t-test. The comparison among four time points was performed using a one-way analysis of variance, followed by the least-significant difference test (LSD-t). The difference was statistically significant when the p-value was less than 0.05.

## RESULTS

### Comparison of puncture time between two groups

The puncture time in the ultrasound-guided group was 3.62±0.83 min, which was significantly shorter than 9.13±1.94 min in the CT-guided group (t=16.923; p<0.001).

### Comparison of numeric rating scale pain score between two groups

Before PRF, there was no significant difference in NRS pain scores between the two groups (p>0.05). At 1, 4, and 12 weeks after PRF, in each group, the NRS pain score was significantly lower than that before PRF, respectively (p<0.05). At each time, the NRS pain score had no significant difference between the two groups (p>0.05) (Table 2).

**Table 2:** Numeric rating scale pain score before and after pulsed radiofrequency in two groups (n=42).

Time	Ultrasound-guided group	CT-guided group	t	p
Before PRF	7.02±0.82	6.92±0.92	0.526	0.600
1 week after PRF	3.54±0.77^a^	3.35±0.91^a^	1.033	0.305
4 weeks after PRF	2.61±0.92^ab^	2.50±0.73^ab^	0.607	0.546
12 weeks after PRF	2.04±1.13^abc^	1.93±1.26^abc^	0.421	0.675
F	247.058	222.248		
P	<0.001	<0.001		

a p<0.05 compared with before pulsed radiofrequency; bp<0.05 compared with 1 week after pulsed radiofrequency; cp<0.05 compared with 4 weeks after pulsed radiofrequency. PRF: pulsed radiofrequency; CT: computed tomography.

### Comparison of effective rate of treatment between two groups

At 12 weeks after PRF, there were 36 effective cases in the ultrasound-guided group, and the effective rate of treatment was 85.71%. There were 34 effective cases in the CT-guided group, and the effective rate of treatment was 80.95%. The effective rate of treatment had no significant difference between the two groups (χ^2^=0.343; p=0.558).

### Comparison of adverse events between two groups

During the intraoperative and postoperative period, there were two cases (4.76%) of hematoma and one case (2.38%) of oculocardiac reflex in the ultrasound-guided group, with nine cases (21.43%) of hematoma and four cases (14.29%) of oculocardiac reflex in the CT-guided group. The incidences of adverse event hematoma and oculocardiac reflex in the ultrasound-guided group were significantly lower than those in the CT-guided group, respectively (χ^2^=5.126, p=0.024; χ^2^=3.896, p=0.048).

## DISCUSSION

PRF for the ophthalmic branch PHN via the foramen ovale trigeminal semilunar ganglion can greatly lessen the pain perception and enhance the patients’ quality of life^12^. This study investigated the efficacy of ultrasound- and CT-guided supraorbital nerve PRF in treating ophthalmic branch PHN. Results showed that, at 1, 4, and 12 weeks after PRF, in the two groups, the NRS pain score was significantly lower than that before PRF, respectively. At 12 weeks after PRF, the effective rate of treatment in the ultrasound- and CT-guided groups was 85.71 and 80.95%, respectively. This indicates that both ultrasound- and CT-guided supraorbital nerve PRFs have good efficacy in treating the ophthalmic branch PHN. This is in line with the findings of the earlier study^7^.

According to PRF theory, a high current density is delivered via the electrode tip. The high-frequency current in a single cycle produces high voltage and heat generation in the target tissue. This current can be administered to the tissue in very short bursts. Because of the relatively long interval between pulses, any heat generated can disperse, preventing the formation of thermal lesions. The high-frequency alternating current produced by PRF acts on the target tissue to cause changes linked to plasticity. This affects the process of central pain pathway on the pain signals and upregulates the expression of c-fos, thus modifying the substance p and endorphin levels and contributing to the long-term analgesic action of PRF by inhibiting pain and encouraging the production and secretion of endogenous opioid peptides^13^. In this study, the high-output voltage PRF was used for treating ophthalmic branch PHN, which was in accordance with similar research^14,15^. The intensity of the electric field is proportional to the square of the voltage. The voltage change indicates a change in the intensity of the electric field when the resistance remains constant. The increased output voltage results in high-energy electric field intensity, which expands the electric field effect, improves the therapeutic effect, and increases the therapeutic range.

Multiple CT images are required for CT-guided puncture to reveal the supraorbital foramen. This is because some individuals have smaller supraorbital foramina, which makes the puncture more challenging. However, in the ultrasound-guided puncture, a lateral-to-medial almost parallel real-time piercing technique is used, which allowed for the clear identification of the supraorbital notch, blood vessels, and nerves^16,17^. In our study, the puncture time in the ultrasound-guided group was significantly shorter than that in the CT-guided group. During the intraoperative and postoperative periods, the incidence of hematoma in the ultrasound-guided group was significantly lower than in the CT-guided group. We attribute this to the advantage of the ultrasound-guided puncture. The ultrasound-guided puncture can accurately avoid the peripheral blood vessels and reduce vascular damage, while the CT-guided puncture is a blind vertical puncture, which may damage peripheral tiny blood vessels. In addition, the incidence of oculocardiac reflex in the ultrasound-guided group was significantly lower than that in the CT-guided group. The reason may be that, compared with the ultrasound-guided puncture, the CT-guided blind vertical puncture may violently stimulate the supraorbital nerve, which may aggravate the oculocardiac reflex.

## CONCLUSION

Both ultrasound- and CT-guided supraorbital nerve PRFs have good efficacy in treating the ophthalmic branch PHN. Compared with the CT-guided PRF, the ultrasound-guided PRF has a faster puncture operation and is safer. It is more worthy of clinical application. However, the patients in two groups were followed up for only 12 weeks, and the longer-time efficacy and safety of these two strategies are not observed. This may be a limitation of our study. In the next, a randomized controlled trial involving a prolonged follow-up is required for obtaining better results.
